# Supplementary material for: Motionless volumetric photoacoustic microscopy with spatially invariant resolution
Source: Nat Commun. 2017 Oct 3;8:780. doi: 10.1038/s41467-017-00856-2 (PMC5626698; doi:10.1038/s41467-017-00856-2)
Supplement: Supplementary file 1 — Supplementary Information [file 41467_2017_856_MOESM1_ESM.pdf]

Supplementary Figures

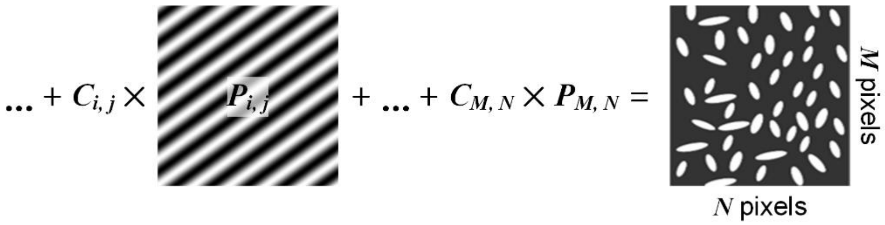

Supplementary Figure 1. Principle of 2D single-detector imaging via Fourier transformation. In Fourier space, an image can be considered as a composition of a series of sinusoidal fringes (that is, $P_{1,1}$, …, $P_{M,\,N}$) with different Fourier coefficients (i.e., $C_{1,1}$, …, $C_{M,\,N}$).

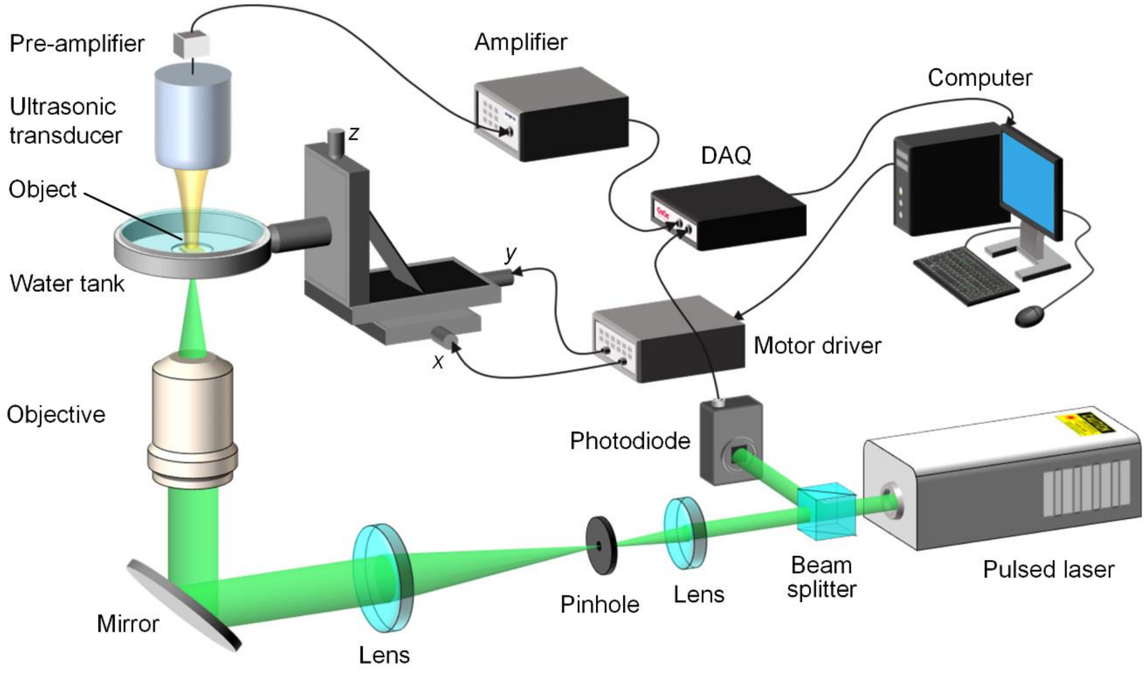

Supplementary Figure 2. Schematic illustration of a conventional PAM system.

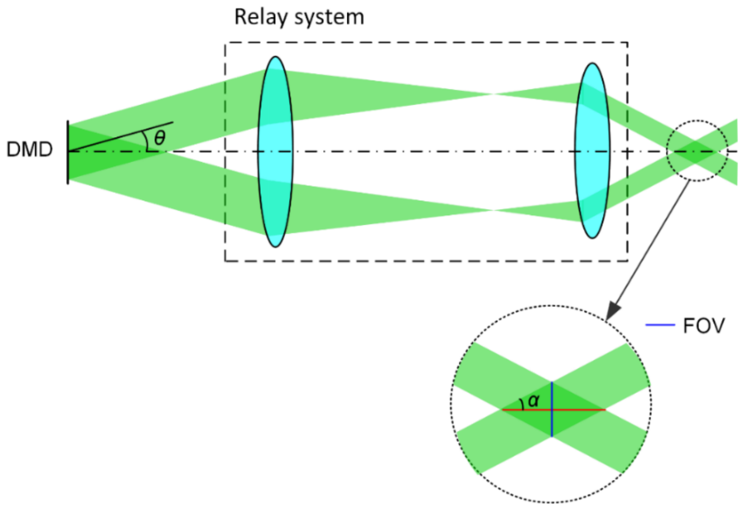

Supplementary Figure 3. FOV of the SIR-PAM system. DMD is used to generate PISFs. θ is the maximum angle of the two collimated beams emerging from the DMD, and α is the maximum angle of the two plane beams incident on the imaging plane. The FOV is decided by the overlap of the two symmetrical collimated beams.

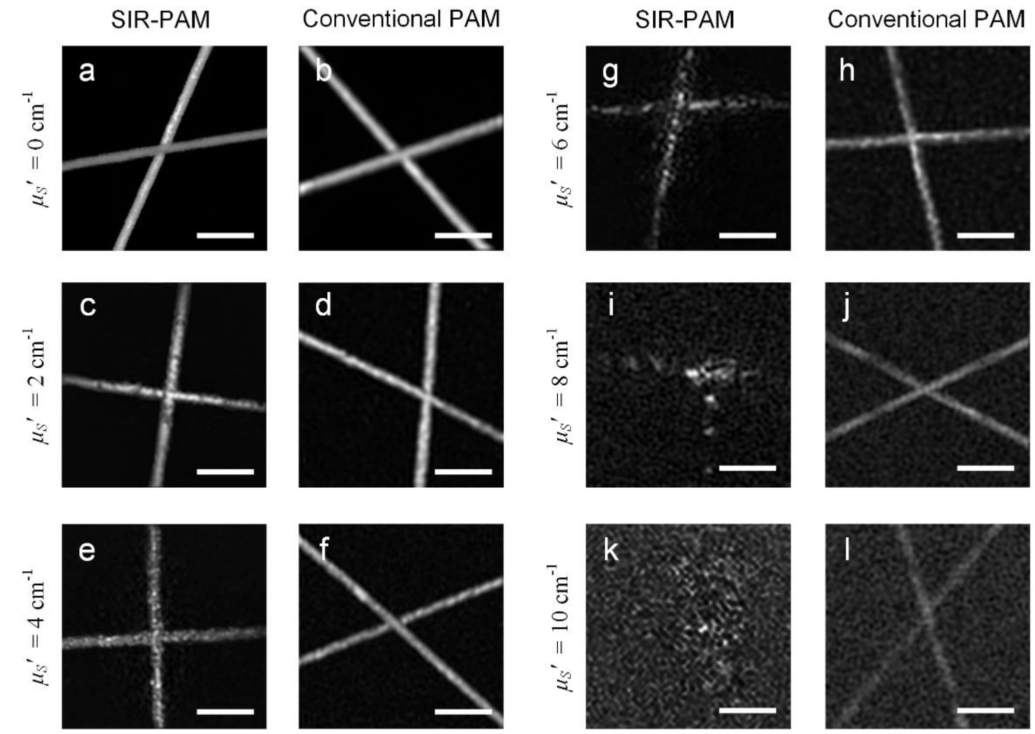

Supplementary Figure 4. Images of crossed fibers embedded in scattering media acquired by SIR-PAM and conventional PAM. The crossed fibers were buried at a 1 mm depth in scattering media with different reduced scattering coefficients μ_s' of = 0, 2, 4, 6, 8 and 10 cm^{-1}. (**a**, **c**, **e**, **g**, **i**, **k**) are the images acquired by SIR-PAM, respectively. While (**b**, **d**, **f**, **h**, **j**, **l**) are the images acquired by conventional PAM, respectively. Scale bars, 50 μm.

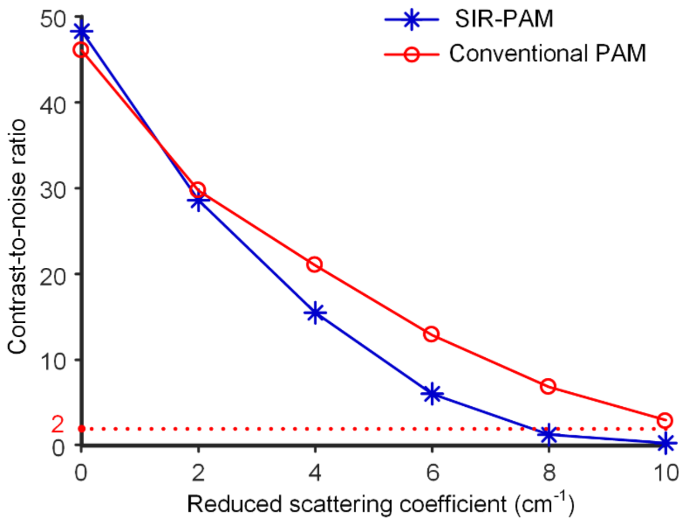

Supplementary Figure 5. Comparison of the contrast-to-noise ratios for conventional PAM and SIR-PAM with respect to different reduced scattering coefficients. The contrast is defined as the mean difference between a crossing fiber signal amplitude and the background amplitude, while the noise is quantified using the standard deviation of the background amplitude.

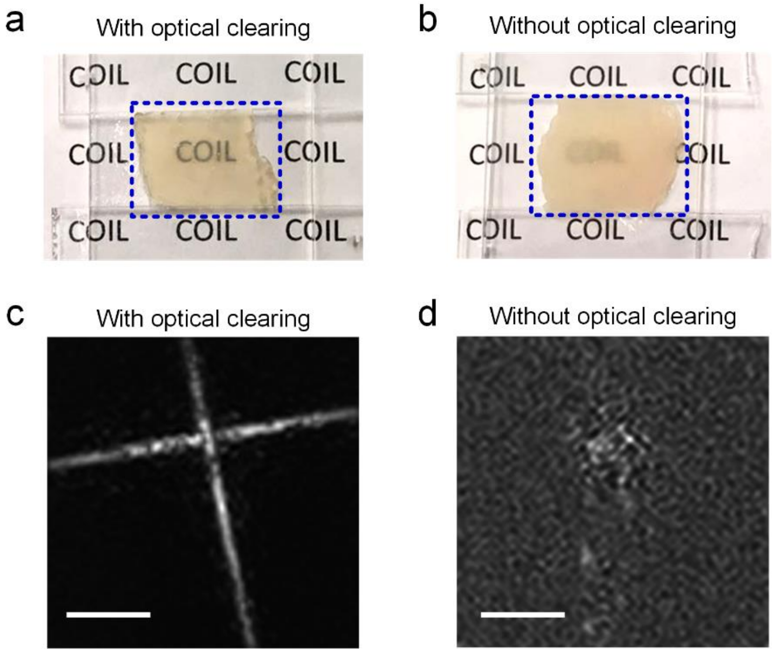

Supplementary Figure 6. Enhancement of imaging depth of SIR-PAM in chicken breast tissue by optical clearing. (**a**, **b**) Photographs of 1 mm thick chicken breast tissue with (**a**) and without (**b**) optical clearing. (**c**, **d**) SIR-PAM images of two crossed carbon fibers beneath 1-mm thick chicken breast tissue with (**c**) and without (**d**) optical clearing. Scale bars, 50 μm.

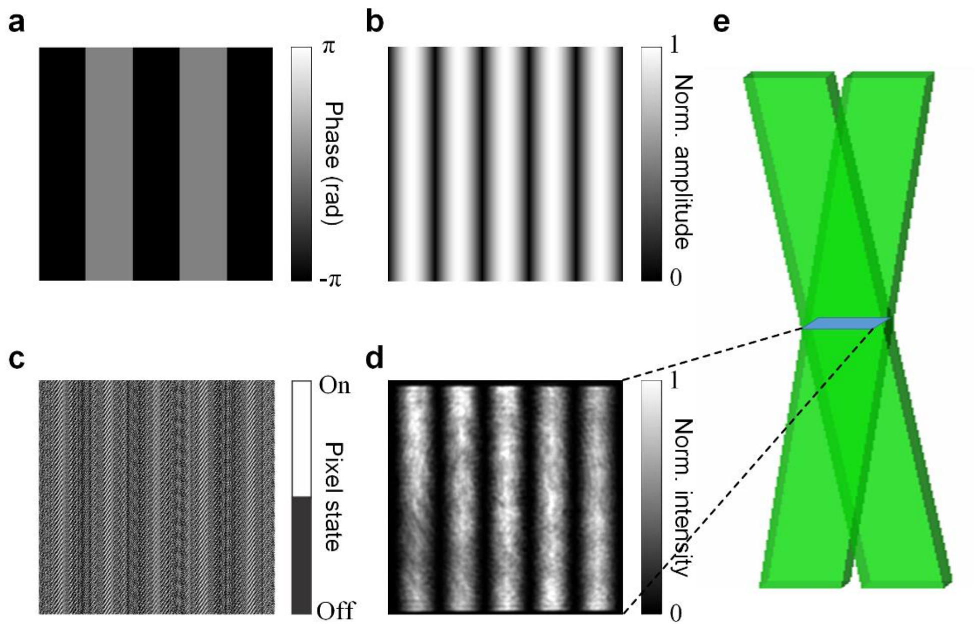

Supplementary Figure 7. Generation of the PISF by complex field encoding with a DMD. The desired phase (**a**) and amplitude (**b**) of the PISF on a given plane can be encoded with a binary hologram (**c**) with the super-pixel method. After the hologram is loaded onto the DMD, the fringe field at z = 0 mm (**d**) is generated when the DMD surface is illuminated by a collimated laser beam. The generated field is propagation invariant and thus a 3D pattern shown in (**e**) is created as long as the two beams overlap.

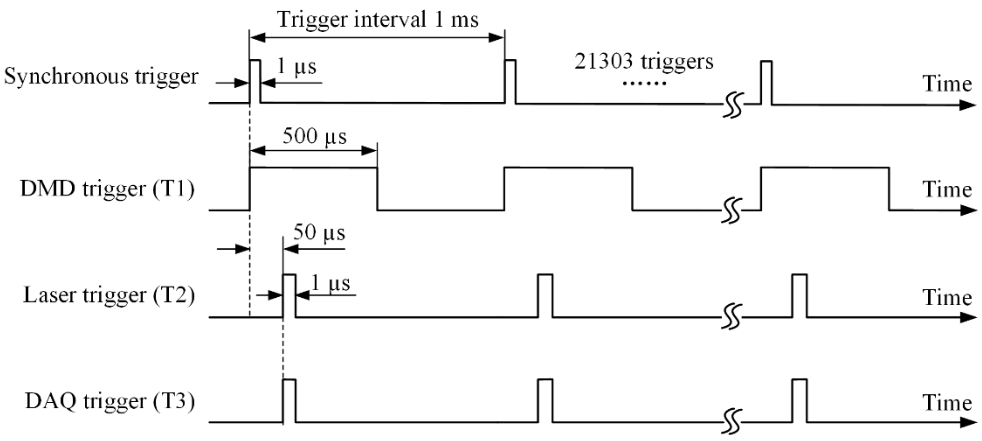

Supplementary Figure 8. Time sequence diagram for triggers of the DMD, laser, and DAQ synchronization. To guarantee the pattern has been refreshed on the DMD when the pulsed laser illuminates, the laser trigger T2 is delayed for 50 μs with respect to the DMD trigger T1. The DAQ trigger T3 is synchronized to T2 with no delay to guarantee synchronous data acquisition from the ultrasonic transducer and the photodiode detector.

Supplementary Notes

Supplementary Note 1: 2D single-detector imaging via Fourier transformation

According to Fourier optics, a 2D image in real space can be considered as a weighted sum of 2D sinusoidal light fringes with different frequencies (Supplementary Figure 1). Actually, the weighted coefficients represent the Fourier spectrum of the image. The Fourier coefficients can be extracted by illuminating the image with phase-shifting sinusoidal fringes and using a single-pixel detector to collect the resulting light. Then, we can reconstruct the image via inverse Fourier transform to the acquired Fourier-spectrum.

Supplementary Note 2: Fourier coefficients extracted from PA signals

For certain spatial frequencies of f_x and f_y, we can acquire three PA signals at depth *z*, $S_0\left(f_x, f_y, z\right)$, $S_{\pi/2}\left(f_x, f_y, z\right)$, and $S_\pi\left(f_x, f_y, z\right)$, according to the illumination sinusoidal fringes with three phases of 0, $\pi/2$, and π. The corresponding Fourier coefficient of the optical absorption distribution $\mu_a\left(x, y, z\right)$ at depth *z* can be calculated as

$$\begin{aligned} C\left(f_x, f_y, z\right) = &\left[S_0\left(f_x, f_y, z\right) - S_\pi\left(f_x, f_y, z\right)\right] \\ &+ i\left[S_0\left(f_x, f_y, z\right) + S_\pi\left(f_x, f_y, z\right) - 2S_{\pi/2}\left(f_x, f_y, z\right)\right], \end{aligned} \tag{1}$$

where i is the imaginary unit.

Supplementary Note 3: Theoretical resolution of SIR-PAM and conventional PAM

Here, we define the theoretical resolution as full-width at half-maximum (FWHM) of the point spread function (PSF). The PSF of SIR-PAM at any depth can be deduced as

$$\mathrm{PSF_{SIR}}\left(x, y\right) = \iint_{\left(\pi f_x\right)^2 + \left(\pi f_y\right)^2 < \left(\mathrm{NA} \cdot 2\pi/\lambda\right)^2} \exp\left[-i\left(2\pi f_x x + 2\pi f_y y\right)\right] \mathrm{d}f_x \mathrm{d}f_y. \tag{2}$$

It can be simplified as $\frac{2\pi^2 \mathrm{NA}}{\lambda r} J_1\left(\frac{4\pi r \mathrm{NA}}{\lambda}\right)$ under polar coordinates $\left(r, \theta\right)$. Thus, its FWHM can be calculated as $0.35\lambda/\mathrm{NA}$ which determines the lateral resolution of SIR-PAM.

In contrast, the PSF of conventional PAM can be written in polar coordinates $\left(r, \theta\right)$ as

$$\mathrm{PSF_{Conv}}\left(r, \theta\right) = \left[\frac{2\mathrm{J}_1\left(2\pi r \cdot \mathrm{NA}/\lambda\right)}{2\pi r \cdot \mathrm{NA}/\lambda}\right]^2. \tag{3}$$

The corresponding FWHM can be calculated as $0.51\lambda/\mathrm{NA}$. Therefore, SIR-PAM has a 1.5-fold finer resolution than conventional PAM.

Supplementary Note 4: Brief description of conventional PAM

To perform the comparative experiments, we established a conventional PAM system as sketched in Supplementary Figure 2. We used a pulsed laser (532 nm wavelength; Elforlight, Ltd.) with a 1 kHz pulse repetition rate as the light source. The pulse energy was monitored by a photodiode detector (SM05PD1A, Thorlabs, Inc.) to compensate for energy fluctuations. The laser beam was expanded and collimated by a beam expander. Instead of being modulated by a DMD, the collimated beam was directly focused by a microscopic objective (NA = 0.1; Olympus, Corp.) to achieve nearly diffraction-limited optical focusing. A PA image was acquired by 2D focal scanning over the entire region of interest, different from the PISF illumination method adopted in SIR-PAM. Here the 2D raster scanning was implemented using two high-resolution translation stages (PLS-85, Physik Instrumente, GmbH & Co. KG). The PA wave was detected by a focused ultrasonic transducer coupled by water, and then amplified by two electronic amplifiers with a combined gain of 48 dB. A data acquisition system (DAQ, ATS9350, Alazar Technologies, Inc.) was used to simultaneously acquire and digitize the PA signal from the amplifier and the laser intensity signal from the photodiode. Then an image of the optical absorption distribution within the object was formed.

Supplementary Note 5: RIAR and FOV of SIR-PAM

In the SIR-PAM system, the square DMD plane with an area of $a \times a$ is relayed to the imaging plane with a scaling factor of β, as shown in Supplementary Figure 3. The effective illumination area $b \times b$ determines the FOV of our imaging system. Thus, we have

$$b = \beta a. \tag{4}$$

In SIR-PAM, the imaged object is illuminated by a series of PISFs, which can be decomposed into two symmetrical collimated beams. The effective numerical aperture (NA) of SIR-PAM system depends on the maximum angle α of the two collimated beams. Here, we employ a super-pixel method to generate the PISFs with a single DMD. Given the maximum angle θ of the collimated beam with respect to the normal direction of the DMD surface, we can write the effective NA approximately as

$$\mathrm{NA} = \sin\alpha \approx \frac{\theta}{\beta}, \tag{5}$$

if the NA is low. In the super-pixel encoding method, $N \times N$ pixels within the DMD are utilized, and the maximum angle θ of the generated plane beams is given by

$$\theta \propto \frac{N\lambda}{a}. \tag{6}$$

According to Supplementary Eqs. 4–6, we have

$$b \times \mathrm{NA} \propto N\lambda. \tag{7}$$

When the pixel number of the DMD is fixed, there is a tradeoff between the FOV and the lateral resolution. If we want to improve them simultaneously, a DMD with more pixels is needed.

In SIR-PAM system, the RIAR is decided by the overlap of the two collimated beams at the maximum incident angle. It is related to the pixel count of the DMD and the NA:

$$\text{RIAR}=\frac{b}{\tan\alpha}\approx\frac{b}{\text{NA}}\propto\frac{N\times\lambda}{\text{NA}^2}. \tag{8}$$

Therefore, we can also improve the RIAR of the system with a higher-resolution DMD for a given NA.

Supplementary Note 6: Study of scattering effect on SIR-PAM

To study how scattering affects the performance of SIR-PAM, we imaged tissue-mimicking phantoms with different scattering coefficients. In the experiments, intralipid (anisotropy g = 0.9) was used as the optical scattering agent, and we mixed it with gelatin-water solution at different concentrations. During this study, the concentrations of these phantoms were set to be 0%, 0.2%, 0.4%, 0.6%, 0.8%, and 1%, which correspond to reduced scattering coefficients μ_s' of = 0, 2, 4, 6, 8 and 10 cm^{-1}, respectively. Two crossed fibers were placed as the imaging targets at a 1 mm depth inside these phantoms. Supplementary Figure 4 shows the imaging results obtained using SIR-PAM and conventional PAM. It can be seen that conventional PAM can resolve the crossed fibers when μ_s' reaches 10 cm^{-1}, while SIR-PAM can resolve the crossed fibers consistently when μ_s' reaches 6 cm^{-1} and partially when μ_s' reaches 8 cm^{-1}. To quantify the visibility, we show the contrast-to-noise ratios (CNR) of the targets in Supplementary Figure 5. If we define the borderline CNR needed to discern the target at a 1 mm depth as 2, the limits of the reduced scattering coefficients for conventional PAM and SIR-PAM are approximately 10 cm^{-1} and 6 – 8 cm^{-1}, respectively. Therefore, SIR-PAM is more susceptible to scattering than conventional PAM.

Supplementary Note 7: SIR-PAM with optical clearing

Even though SIR-PAM is more susceptible to optical scattering than conventional PAM, optical clearing can significantly suppress the influence of scattering and help take full advantage of SIR-PAM. Here we experimentally demonstrated the enhancement of imaging depth in biological tissue with the help of optical clearing. Glycerol (G33-1, Fisher Scientific, Inc.) was used as the optical clearing agent, which can be used both *in vitro* and *in vivo*[1]. Supplementary Figures 6 (a) and (b) show the 1 mm chicken breast tissue with and without optical clearing. It can be seen that the chicken breast tissue became much more transparent after optical clearing. Then two carbon fibers underlying the chicken breast tissue were imaged with and without the help of optical clearing, respectively. As shown in Supplementary Figures 6 (c) and (d), SIR-PAM clearly resolved the crossed fibers under the scattering tissue with optical clearing. Therefore, optical clearing can help SIR-PAM image deeper in highly scattering media.

Supplementary Note 8: Number of the PISFs required

Limited by the numerical aperture (NA) of the objective,

$$(\pi f_x)^2 + (\pi f_y)^2 < (\mathrm{NA} \times 2\pi/\lambda)^2, \tag{9}$$

where λ is the optical wavelength. According to discrete 2D Fourier transformation, the values of f_x and f_y meet the relationship of

$$\begin{cases} f_x \cdot L = m, & (m = \pm 1, \pm 2, \cdots \pm M) \\ f_y \cdot L = n, & (n = \pm 1, \pm 2, \cdots \pm N) \end{cases}, \tag{10}$$

where $L \times L$ is the square illumination area of the sinusoidal fringes. According to Supplementary Eqs. 9 and 10, the number of effective Fourier coefficients is definitive. Then, the number of PISFs that are used to retrieve the Fourier spectrum can be calculated.

For example, if the illumination area is 180×180 μm^2, the NA of the objective is 0.1, and the wavelength is 532 nm, the number of acquired Fourier coefficients is 14202. Utilizing the symmetry of the Fourier coefficients, we need to acquire only half of the coefficients. In the experiment, we use the three-step phase shifting method to derive the Fourier coefficients. Therefore, the total number of PISFs required is 21303.

Supplementary Reference

1 Zhou, Y., Yao, J. & Wang, L. V. Optical clearing-aided photoacoustic microscopy with enhanced resolution and imaging depth. *Opt. Lett.* **38**, 2592-2595 (2013).
