# Supplementary material for: Motionless volumetric photoacoustic microscopy with spatially invariant resolution
Source: Nat Commun. 2017 Oct 3;8:780. doi: 10.1038/s41467-017-00856-2 (PMC5626698; doi:10.1038/s41467-017-00856-2)
Supplement: Supplementary file 3 — Description of Additional Supplementary Files [file 41467_2017_856_MOESM3_ESM.pdf]

Description of Additional Supplementary Files

File Name: Supplementary Movie 1
Description: Animation of our proposed motionless volumetric spatially invariant resolution photoacoustic microscopy.

File Name: Supplementary Movie 2
Description: Volume-rendered images of carbon fibers.

File Name: Supplementary Movie 3
Description: Volumetric imaging of zebrafish larvae *in vivo*.
